# Diagnosis of *Fusarium oxysporum* f. sp. *ciceris* causing *Fusarium* wilt of chickpea using loop-mediated isothermal amplification (LAMP) and conventional end-point PCR

**DOI:** 10.1038/s41598-023-29730-6

**Published:** 2023-02-14

**Authors:** Saidi R. Achari, Ross C. Mann, Mamta Sharma, Jacqueline Edwards

**Affiliations:** 1 AgriBio, Agriculture Victoria Research, DJPR, Bundoora, VIC Australia; 2 School of Applied Systems Biology, La Trobe University, Bundoora, VIC Australia; 3 International Crops Research Institute for the Semi-Arid Tropics (ICRISAT), Patancheru, Hyderabad, India

**Keywords:** Genetics, Molecular biology

## Abstract

*Fusarium oxysporum* (Fo) is ubiquitous in soil and forms a species complex of pathogenic and putatively non-pathogenic strains. Pathogenic strains cause disease in over 150 plant species. *Fusarium oxysporum* f. sp. *ciceris* (Foc) is a major fungal pathogen causing *Fusarium* wilt in chickpeas (*Cicer arietinum*). In some countries such as Australia, Foc is a high-priority pest of biosecurity concern. Specific, sensitive, robust and rapid diagnostic assays are essential for effective disease management on the farm and serve as an effective biosecurity control measure. We developed and validated a novel and highly specific PCR and a LAMP assay for detecting the Indian Foc race 1 based on a putative effector gene uniquely present in its genome. These assays were assessed against 39 Fo *formae speciales* and found to be specific, only amplifying the target species, in a portable real-time fluorometer (Genie III) and qPCR machine in under 13 min with an anneal derivative temperature ranging from 87.7 to 88.3 °C. The LAMP assay is sensitive to low levels of target DNA (> 0.009 ng/µl). The expected PCR product size is 143 bp. The LAMP assay developed in this study was simple, fast, sensitive and specific and could be explored for other Foc races due to the uniqueness of this marker to the Foc genome.

## Introduction

Chickpea (*Cicer arietinum*) is an economically important crop worldwide and plays an important nutritional role in the diets of millions of people, especially in developing countries, providing an essential source of protein, calcium, iron, phosphorus, and other minerals^1^. It is the second-largest cultivated legume crop after dry beans (*Phaseolus vulgaris*) globally^2^. It is grown throughout tropical, subtropical and temperate regions in South and West Asia, East and North Africa, southern Europe, North and South America, and Australia^2^. Asian countries contribute to 83% of global chickpea production. Approximately 12 million tonnes of chickpea are produced annually, with India contributing approximately 64%, followed by Australia holding 7% of the global share^1^. In 2021, approximately 500,000 Ha was planted in Australia, producing 732,000 tonnes. Most grains were exported, fetching AUS$543 million in foreign earnings^3^.

Global chickpea production is highly dependent on various biotic and abiotic stresses. One of the critical biotic stresses, *Fusarium* wilt, causes significant economic losses ranging from 10 to 40% in many countries but has the potential to cause complete crop loss under disease-favourable conditions^4–7^. It is caused by the soil and seed-borne fungal pathogen *Fusarium oxysporum* (Fo) *forma specialis* (f. sp.) *ciceris* (Foc). *Fusarium oxysporum* f. sp. *ciceris* is a member of the Fo species complex (FOSC), a soil-borne fungus comprising pathogenic and putatively non-pathogenic strains. Plant pathogenic Fo strains cause vascular wilt and cortical rot in many agricultural crop species. They are classified into host-specific forms (*formae speciales*, ff. spp.) and are often further subdivided into races based on their capacity to infect different cultivars of a plant species.

*Fusarium* wilt in chickpea was initially reported in India by Butler in 1918, but its aetiology was not correctly determined until 1940 by Padwick^8^. It is widespread in most chickpea-growing areas of Asia, Africa, southern Europe and the Americas but is considered to be absent from Australia^9^. Eight physiological races (0, 1A, 1B/C, 2, 3, 4, 5 and 6) based on disease reactions on 10 chickpea differential cultivars have been reported globally in Foc^10^. There are two pathotypes based on aboveground symptoms: one causing yellowing and the other causing wilting^11,12^. Races 1A, 2, 3, 4, 5 and 6 induce wilting symptoms, including severe chlorosis and flaccidity combined with vascular discolouration followed by plant death. Races 0 and 1B/C are less virulent than the other races and induce yellowing symptoms^11,13^. The eight races have distinct geographic distributions, with race 1A more widely distributed across India, Mediterranean regions and California^14^. Races 2, 3 and 4 are found in India and Ethiopia^15–17^ while races 0, 1B/C, 5 and 6 are found mainly in the Mediterranean regions and the USA^16,18,19^.

Due to high genetic variation in the Foc population in India, Dubey et al.^20^ in 2012 developed a new set of chickpea differential cultivars to identify the Indian Foc population and characterised them into eight new races (races 1–8). How these newly Indian characterised races compare to the globally characterised races is not yet known. Isolates belonging to the eight Indian races were found to belong to the same vegetative compatibility group (VCG)^21^. Vegetative compatibility grouping is a system for genetic diversity analysis and classification, as proposed by Puhalla^22^. Isolates collected globally, irrespective of geographical distribution, race and symptom type, were also found to be from a single VCG^23^. Isolates belonging to the same VCG can exchange genetic information via heterokaryosis and a parasexual cycle^24^, hence are clonally related and genetically similar.

Disease symptoms can develop at any stage of plant growth and affected plants may be grouped in patches or appear spread across the field^8,25^. Disease symptoms in highly susceptible cultivars can develop 25 days after sowing, including flaccidity of individual leaves followed by a dull-green discolouration, desiccation and collapse of the whole plant (designated ‘early wilt’)^8^. The symptoms are usually more conspicuous at the onset of flowering, 6–8 weeks after sowing and can also appear up to the podding stage (‘late wilt’)^26^. Late wilted plants exhibit drooping of the petioles, rachis and leaflets, followed by yellowing and foliage necrosis^26^. Initially, drooping is observed in the upper part of the plant, but within a few days, the entire plant collapses^26^. Roots and stems of affected plants develop a dark-brown discoloration of xylem tissues visible upon vertical split or when cross-sectioned^26^.

Molecular diagnostic assays for Foc are highly desirable as the determination of the organism to the taxonomic level of f. sp. using nonmolecular methods is costly in terms of time and resources. However, horizontal gene transfer between strains in the FOSC has resulted in a polyphyletic origin of host specificity in most ff. spp., which prevents molecular identification of strains based on conserved, vertically inherited genes^27^. Pathogenic strains may share higher sequence similarity of conserved genes with putatively non-pathogenic strains or other ff. spp.

Currently, two studies have developed molecular markers for diagnostics of Foc. One is a loop-mediated isothermal amplification (LAMP) assay based on a conserved gene, *translation elongation factor 1-alpha* (EF1α)^28^. The aim was to discriminate Fo from other fungal pathogens that infect chickpeas and therefore, the assay is not specific to Foc but distinguishes Fo from other fungi. The other is a conventional PCR assay based on random amplified polymorphic DNA (RAPD) fragments, resulting in sequence characterized amplified region (SCAR) markers^29^. SCAR markers are suboptimal for f. sp. discrimination because they are based on genomic regions that are not necessarily required for virulence. Furthermore, as they can be localized anywhere on the genome, there is often little to no sequence data available in the public databases for comparison with other sequences. With the amount of genomic data currently available in the public domain, it has been determined that the markers used by Jiménez-Gasco and Jiménez-Díaz^29^ to identify Foc races 0, 1A, 5 and 6 contain a fragment that is identical to the *Impala* transposon^30^. The impala elements in strains of different ff. spp. are highly similar^31^. Molecular markers based on such regions could produce false-positive results.

Effectors are functional elements in the pathogen-host interaction and have shown very limited sequence diversity between strains of the same f. sp., making them potential markers for host-specific pathogenicity^32^. Effector proteins in Fo and in other fungal pathogens may have pathogenicity roles and act as enzymes, toxins, transcription factors, elicitors or virulence factors^33^. One of the main groups of effector genes in Fo encodes for proteins that are secreted in xylem (SIX1-SIX14)^34–37^, but the pathogenicity role has been experimentally validated for only a few members of that group^38,39^. Recent studies have shown that host specificity in Fo is governed by the suite of effector genes^33,40^. Both presence-absence polymorphisms and the individual effector gene sequence predict a strain’s host range^32,33^. These genes, therefore, should be used as molecular markers for the discrimination of ff. spp. within the FOSC. Effectors have successfully been used as molecular markers within the FOSC to discriminate Fo f. sp. *lycopersici*^41^ and *Cucurbitaceae*-affecting strains^32^ from other ff. spp. New Fo genomes are being constantly assembled and made publicly available, making whole-genome comparative studies the new gold standard for identifying molecular markers for diagnostics.

The spread of Foc race 1 in India is not known; however, it has been confirmed to be present in Andhra Pradesh and Karnataka states^20,21^. Indian Foc race 1 has been more intensively studied than any other races. These studies have identified the molecular mechanisms behind the chickpea-Foc1 interactions^42^ and have used Foc race 1 for comparative genomics studies to identify conditionally dispensable sequences in legume-infecting Fo ff. spp.^43^. These studies all used Foc race 1 strain 38-1 obtained from ICRISAT, India. Therefore, a well-curated genomic sequence for strain 38-1 exists on the NCBI GenBank and single-spored cultures of Foc race 1 strain 38-1 are maintained at ICRISAT, India. Genomic DNA of Foc race 1 strain 38-1 was imported from ICRISAT, India, specifically for use in this study.

The aim of this study was to utilise a comparative genomics approach through the use of OrthoFinder^44^ to identify an effector gene unique to Indian Foc race 1 and to develop a specific, sensitive and rapid LAMP and a PCR assay that can selectively discriminate isolates of Indian Foc race 1 from other FOSC ff. spp.

## Materials and methods

### Fungal isolates and genomes

We created a database of 356 Fo genomes of 39 ff. spp. of exotic and endemic isolates. One hundred and fifty genomes of 33 ff. spp. were retrieved from the NCBI GenBank. These ff. spp., with the number of isolates in parentheses, are: *albedinis* (1), *apii* (3), *capsici* (1), *cepae* (3), *ciceris* (1), *conglutinans* (6), *coriandrii* (2), *cubense* (6), *cucumerinum* (9), *fragariae* (19), *gladioli* (3), *koae* (1), *lagenariae* (4), *lilii* (1), *lini* (5), *luffae* (2), *lycopersici* (8), *matthiolae* (1), *medicaginis* (1), *melongenae* (2), *melonis* (10), *momordicae* (2), *mori* (1), *narcissi* (2), *nicotianae* (4), *niveum* (12), *pisi* (1), *radicis-cucumerinum* (3), *radicis-lycopersici* (1), *raphani* (1), *sesami* (1), *spinaciae* (10), *tulipae* (1), *vasinfectum* (10) (Supplementary Table 1). An additional 12 genomes only identified as Fo were included; four of these 12 genomes were of non-pathogenic Fo isolated from chickpeas from Ethiopia (Supplementary Table 1).

Two hundred and six cultures of Fo already present in Australia (henceforth called endemic) were sourced from culture collections across the country (Victorian Plant Pathogen Herbarium (VPRI), Royal Botanic Gardens (RBG) Sydney, NSW Plant Pathology and Mycology Herbarium (DAR), NSW Department of Primary Industries (DPI), Melbourne University and South Australia Research and Development Institute (SARDI)), and their genomes generated to add to the database. One hundred and four isolates were sequenced and assembled as part of this study, while genomes of other isolates were generated as part of a previous study^27^. Seventy of these isolates are of Fo isolated from diseased horticultural plants, 14 isolates are from soil from the natural ecosystems and 122 isolates are of 17 ff. spp.. These ff. spp., with the number of isolates in parentheses, are *basilici* (5), *canariensis* (13), *conglutinans* (1), *cubense* (5), *dianthi* (1), *fragariae* (3), *lycopersici* (9), *medicaginis* (2), *melonis* (9), *niveum* (7), *passiflorae* (5), *pisi* (35), *spinaciae* (1), *tracheiphilum* (1), *tulipae* (4), *vasinfectum* (17) and *zingiberi* (4) (Supplementary Table 1). Purified Foc race 1 genomic DNA of strain 38–1 was sourced from ICRISAT, India, to be used as a positive control and the genome was sequenced and assembled as part of this study.

### Culture growth and DNA extraction

Endemic Fo cultures were single-spored using the method described by Burgess et al.^45^ and cultures were grown and mycelia were harvested as described by Achari et al.^27^. Genomic DNA was extracted using Wizard® Genomic DNA Purification Kit (www.promega.com). The quality of the genomic DNA was assessed using a NanoDrop ND-1000 spectrophotometer (NanoDrop Technologies). DNA samples with 260/280 ratios of ~ 1.8 were used for downstream library preparation.

### Sequencing and genome assembly

Paired-end libraries were prepared for endemic isolates and Foc genomic DNA using the Illumina Nextera XT DNA library prep kit according to the manufacturer’s protocols. These libraries were sequenced using Illumina NovaSeq 6000. Fastq sequence files generated from the sequencing run were filtered using fastp, filtering sequences based on a minimum length of 50 bp and removal of adaptors^46^. Low-quality reads (< Q20) from the fastq sequence files were also filtered using fastp^46^. The reads were de novo assembled using SPAdes version 3.7.1^47^. Contigs < 5X coverage and < 200 bp were removed. The quality and completeness of the assembled genomes were measured using BUSCO as described by Achari, et al.^27^ and assemblies having greater than 97% of 3725 core Sordariomycete genes as estimated by BUSCO were kept in the database for comparative genomic analysis.

### Identification of the molecular marker

All the genomes in the database were annotated using Augustus^48^. The amino acid sequences of each genome were used for comparative genomic analysis using OrthoFinder^44^. The OrthoFinder divided the 356 genomes into orthogroups. An orthogroup is a gene family or clade of genes defined at a specific taxonomic level^44^. These orthogroups were assessed across the 356 genomes and a single orthogroup unique to Foc was identified as the Foc molecular marker. The gene number of the Foc-specific orthogroup was used to retrieve the amino acid and nucleotide sequence from the Foc genome gff file.

### LAMP and PCR primer design

**Figure 1:**
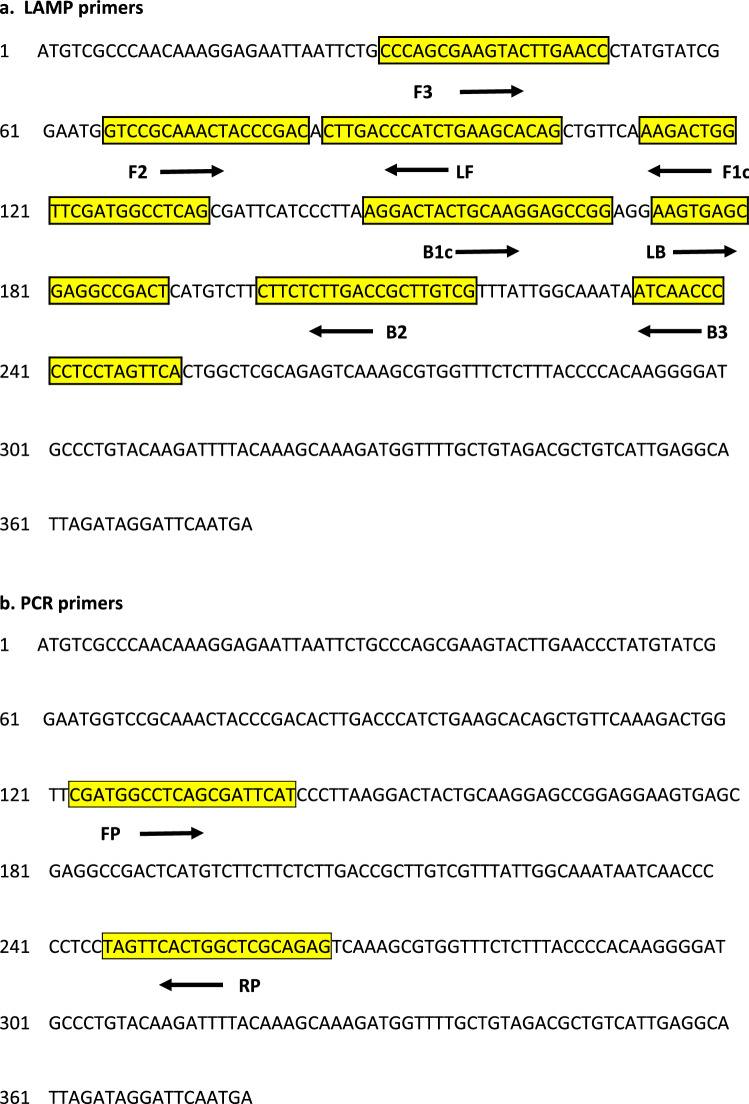
Schematic representation of position and sequence of the designed primer sets within the nucleotide sequence of the putative effector gene of *Fusarium oxysporum* f. sp. *ciceris* used for (**a**) LAMP and (**b**) PCR assays.

Novel LAMP and end-point conventional PCR primers were designed targeting the Foc unique molecular marker. PCR and LAMP primers were designed and analysed using Primer 3^49^ in Geneious and Primer Explorer V5 software (http://primerexplorer.jp), respectively. The LAMP primers included: two outer primers (forward primer, F3; backward primer, B3), two inner primers (forward inner primer, FIP (F1c and F2); backward inner primer, BIP (B1c and B2) and two loop primers (forward loop primer, FL; backward loop primer, BL). The specificity of the primers was further tested using the NCBI Primer-BLAST on Refseq mRNA against *Fusarium* as an organism. Primers for both LAMP and PCR were synthesized by Merck (https://www.sigmaaldrich.com). Primer sequences and their relative positions on the Foc molecular marker gene are shown in Table 1 and Fig. 1, respectively.

**Table 1:** Details of the primers designed based on the putative effector gene found to be unique to *Fusarium oxysporum* f. sp. *ciceris*.

Primer type	Primer	Length of primer (bp)	Sequence (5′-3′)
LAMP-Foc	F3	20	CCCAGCGAAGTACTTGAACC
	B3	20	TGAACTAGGAGGGGGTTGAT
	FIP (F1c and F2)	42	CTGAGGCCATCGAACCAGTCTTGTCCGCAAACTACCCGAC
	BIP (B1c and B2)	41	AGGACTACTGCAAGGAGCCGGCGACAAGCGGTCAAGAGAAG
	LF	20	CTGTGCTTCAGATGGGTCAAG
	LB	18	AAGTGAGCGAGGCCGACT
Conventional end-point PCR			
Foc	Forward (FP)	20	CGATGGCCTCAGCGATTCAT
	Reverse (RP)	20	CTCTGCGAGCCAGTGAACTA
ITS	ITS1	19	TCCGTAGGTGAACCTGCGG
	ITS4	20	TCCTCCGCTTATTGATATGC

### Development of the LAMP assay

The primer ratio (F3/B3: FIP/BIP: LF/LB) used for the assay was 1:8:2 with the final concentrations of 0.2 µM, 1.6 µM and 0.4 µM for F3/B3, FIP/BIP and LF/LB. LAMP reactions were performed and optimised in the Genie III triplicates and repeated three times. The assay was run using WarmStart^®^ LAMP Kit (DNA & RNA) (New England BioLabs Inc.) in a 25 μL reaction volume. The components of the reaction were 12.5 μL of LAMP 2X Master Mix, 0.5 μl Fluorescent dye (50X), 2.5 μl LAMP Primer Mix (10X), 8.5 μl water and 2 μl (5 ng/ul) DNA. 2 μL of ultrapure water was used as no template control (NTC). LAMP amplification reactions were run at 65 °C for 30 min, followed by an annealing analysis from 95 to 60 °C with ramping at 0.05 °C per second that allowed the generation of derivative melting curves. The approximate running time was 40 min.

Upon completion of the run, the amplification and anneal derivative curves were visualised on the Genie III screen to ensure amplification occurred as expected. No amplification supported by flat amplification lines are expected from non-target species and NTCs. The time to amplification (minutes) and anneal derivative temperature (°C) were recorded from the results table. The run details such as the date and the run number of each assay completed on the Genie III were recorded for ease of run file transfer and analysis using a PC version of the software Genie Explorer version V2.0.6.3. The blue channel of the Genie III was used for visualising LAMP assays in this study.

### Specificity of detection of the LAMP assay

When designing an assay, it is imperative to test close relatives of the target pathogen to ensure cross-reactions do not cause a false positive result. The LAMP specificity assay was performed with template fungal DNA from 17 other Fo ff. spp. (*basilici*, *canariensis*, *conglutinans*, *cubense*, *dianthi*, *fragariae*, *lycopersici*, *medicaginis*, *melonis*, *niveum*, *passiflorae*, *pisi*, *spinaciae*, *tracheiphilum*, *tulipae*, *vasinfectum* and *zingiberi*). Only a single isolate per f. sp. was used for specificity testing. The reaction was run in triplicate and the experiment was repeated twice. Since there were more than eight samples, this assay was run on a qPCR machine using the same assay conditions described previously for the Genie III run.

Once the run was completed, the amplification and anneal derivative curves were visualised on the qPCR screen to ensure that amplification had occurred as expected. As described above, non-target species and NTCs are expected to have flat amplification lines. The time of amplification (minutes) and anneal derivative temperature (°C) were recorded from the results table. The eds file of the run was transferred and analysed using a PC version of QuantStudio Design and Analysis software.

### Sensitivity of the LAMP assay

A five-fold serial dilution of genomic Foc DNA was prepared using ultrapure water. Starting DNA concentration was quantified using a Quantus Fluorometer (www.promega.com). The DNA was serially diluted using ultrapure water from 9 to 0.00009 ng/µl (1:1 to 1:100,000). The sensitivity of the LAMP assay was tested using the serially diluted DNA on a qPCR machine. The experiment was run twice in triplicates.

### Conventional polymerase chain reaction (PCR) assay

PCR was executed using Veriti (Applied Biosystems, ThermoFisher Scientific) in a 25 µl reaction volume which included the following components: 12.5 µl of Hot Start Taq 2X Master Mix (New England Biolabs), 1.25 µl of forward and reverse primers, 2 µl (10 ng) of template DNA and 8 µl of ultrapure water. The Foc-specific PCR was screened against 17 other ff. spp. (*basilici*, *canariensis*, *conglutinans*, *cubense*, *dianthi*, *fragariae*, *lycopersici*, *medicaginis*, *melonis*, *niveum, passiflorae*, *pisi*, *spinaciae*, *tracheiphilum*, *tulipae*, *vasinfectum* and *zingiberi*). Only one isolate per f. sp. was used for PCR*.* Ultrapure water was used as an NTC. Different cycling PCR parameters were trialled to identify the optimum PCR cycling conditions. After amplification, 3 μl of gel loading dye was added to each sample and resolved on 2% agarose gel in 0.5X TBE buffer. The gel electrophoresis was run at 100 V for an hour. The size of amplified DNA fragments was estimated with a hyperladder™ 100 bp (Bioline). The expected amplicon size was 143 bp. Internal transcribed spacer (ITS) universal primers ITS1/ITS4, designed to amplify a ribosomal DNA fragment of approximately 544 bp was used as an internal control for Fo.

## Results

### Identification of Foc-specific molecular marker

The molecular marker identified for detection of Foc race 1 is a 378 bp gene which encodes for a protein of 125 amino acids in length. Specificity of the gene to the Foc genome was confirmed through in silico and in vitro experimentations. In silico analysis was carried out using BLASTn and BLASTx with default settings against the nucleotide and protein databases on the NCBI GenBank and using BLASTn (evalue: 1E-10) against the genomes in the database created in this study. Specificity testing on LAMP and PCR assay has shown that this gene was found to be uniquely present in the Foc genome. This gene was confirmed as an effector gene using EffectorP versions 1.0, 2.0 and 3.0 (https://effectorp.csiro.au/)^50–52^ with default settings.

### Specificity and sensitivity of Foc race 1 LAMP assay

**Figure 2:**
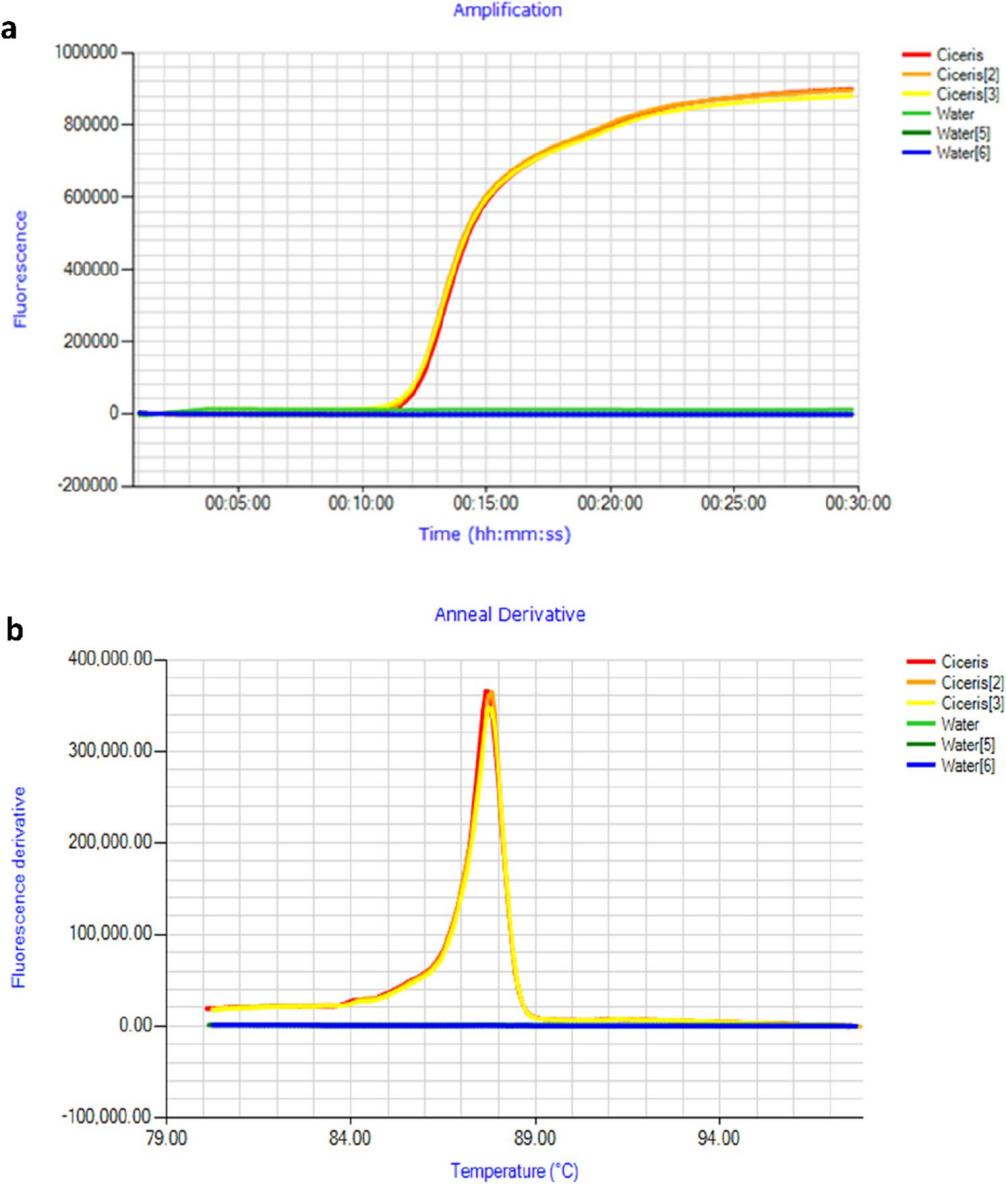
Optimised LAMP assay performed on genomic *Fusarium oxysporum* f. sp. c*iceris* DNA (**a**) Amplification profile, with positive samples amplifying in < 13 min. (**b**) Anneal derivative of LAMP amplicons, with an average anneal derivative of 87.7 °C.

The Foc race 1 LAMP assay on Genie III produced amplification of the target f. sp. DNA in less than 13 min with an average anneal derivative temperature of 87.7 °C (Fig. 2). There was no amplification of the NTC. The LAMP specificity assay was performed with template fungal DNA from 17 other Fo ff. spp. (*basilici*, *canariensis*, conglutinans, *cubense*, *dianthi*, *fragariae*, *lycopersici*, *medicaginis*, *melonis*, *niveum, passiflorae*, *pisi*, *spinaciae*, *tracheiphilum*, *tulipae*, *vasinfectum* and *zingiberi*). At optimum conditions, no positive amplification was observed in the case of other ff. spp. The average time to amplify three replicates of Foc genomic DNA on a qPCR machine was 10 min and 51 s, with an average anneal derivative temperature of 88.3 °C.

A five-fold serial dilution of genomic Foc DNA was prepared using ultrapure water. We successfully detected amplification of all the replicates until the three-fold dilution, up to 0.009 ng/µl of DNA. Only one replicate was amplified for samples with 0.0009 ng/µl of DNA, while there was no amplification from any replicates with 0.00009 ng/µl of DNA. The amplification time became slower in a predictable manner as DNA template concentrations reduced, showing a strong relationship between increased amplification times and decreasing DNA concentrations (Table 2).

**Table 2:** The average amplification time and anneal derivative for serial dilutions of *Fusarium oxysporum* f. sp. *ciceris* genomic DNA.

DNA concentration	Average time to amplification (m:s)	Average anneal derivative (°C)
9 × 10^−1^	11:36	88.2
9 × 10^−2^	12:19	88.1
9 × 10^−3^	16:06	88.0

### Detection of Foc race 1 using PCR

**Figure 3:**
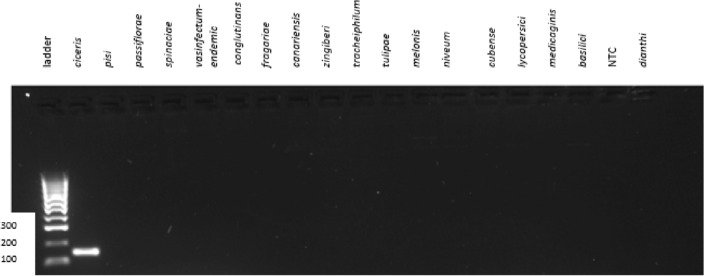
Agarose gel showing amplification products from PCR using genomic *Fusarium oxysporum* f. sp. c*iceris* (Foc) DNA. Lane 2 with Foc DNA amplified showing a product size of 143 bp. There is no amplification from any other ff. spp.

The optimum cycling parameters were initial denaturing for 30 s at 95 °C; 28 cycles of [denaturing at 95 °C for 30 s, annealing at 60 °C for 30 s, extension at 72 °C for 30 s] and final extension at 72 °C for 1 min. The primer pair Foc-FP/Foc-RP amplified a unique DNA fragment of approximately 143 bp in Foc race 1 but did not yield amplification products for any of the 17 other ff. spp. (Fig. 3). Internal transcribed spacer (ITS) universal primers ITS1/ITS4, designed to amplify a ribosomal DNA fragment of approximately 544 bp, yielded positive PCR reactions for all the isolates (data not shown).

## Discussion

Loop-mediated isothermal amplification (LAMP) is becoming a reliable and robust method for detecting and identifying a variety of phytopathogens. Isothermal amplification with easy detection of amplifications makes LAMP a simple-to-operate and easy-to-read molecular diagnostic tool for laboratory and in-field settings. Several LAMP-based diagnostic kits and assays have been developed targeting a range of pathogens^53^. Many technical advances have been made over the years to satisfy the demands of the molecular diagnostic industry for more specificity, sensitivity, efficiency, and rapidity in diagnostics^54^.

*Fusarium oxysporum* f. sp. *ciceris* is a high-priority exotic grain pest of biosecurity importance to Australia. Specific, sensitive, robust and rapid identification of Foc is necessary for effective biosecurity control. Effector genes are known to be involved in pathogenicity and determine host-specificity. We used a comparative genomics approach to comprehensively screen 356 Fo genomes of 39 ff. spp. to identify a putative effector gene uniquely present in Foc race 1. PCR and LAMP assays were developed that were very specific and sensitive to Indian Foc race 1.

Two previous Foc diagnostic assays have been published. A LAMP assay was developed based on the *translation elongation factor* (TEF) gene^28^. This assay is not specific to Foc but distinguishes Fo amongst other pathogens causing diseases on chickpea plants. If a soil sample is screened for the presence of Foc before planting chickpeas, this assay will produce a positive amplification irrespective of Foc being absent because Fo is ubiquitously present in the soil as non-pathogenic strains, and the assay is generic to Fo and not specific to Foc.

A Foc PCR diagnostic assay was developed in 2003 based on an impala transposon^29,30^ and is the test endorsed for use in Australia’s NDP36, the National Diagnostic Protocol for *Fusarium oxysporum* f. sp. *ciceris*^9^. However, this assay was not comprehensively screened against other ff. spp.^29^. There are five subfamilies of impala transposon identified in Fo, and most of these are present in multiple copies in several strains with different host specificities^55^. Using an impala transposon as a molecular marker may lead to unspecific amplifications and consequently a false positive diagnosis. Genome sequences of all ff. spp. obtained from NCBI GenBank had an incomplete transposon sequence except for the two strains of f. sp. *lini* (F282- JABJUB010000001.1 and F329- JABJUE010000001.1). The sequence region where the forward and reverse primers bind was present in two pieces in the genomic sequence, with the middle sequence region missing (Supplementary Fig. 2). The forward and reverse primers and the f. sp. *lini* sequences showed no nucleotide sequence difference at the binding sites (Supplementary Fig. 2). Since the transposon sequence was incomplete for most of the ff. spp., we ran the PCR assay against some ff. spp. and found the assay not to be specific to Foc. Their assay will amplify isolates belonging to ff. spp.: *lycopersici*, *tulipae*, *canariensis*, *medicaginis*, *passiflorae*, *tracheiphilum*, *pisi*-race 5, *dianthi* and *vasinfectum* (Australian endemic strains) (Supplementary Fig. 3) and *lini* (Supplementary Fig. 2).

Australian biosecurity measures restrict the importation of live cultures of exotic plant pathogens of biosecurity significance to Australia. We were only able to source genomic DNA of Foc race 1 strain 38-1 from ICRISAT, India, and this served as a model for all of the Foc races. Our Foc molecular marker has been comprehensively screened against 39 ff. spp. as well as endemic Fo present in natural ecosystems in Australia and non-pathogenic Fo isolated from chickpea plants. We were unable to in silico test these assays on other Foc races as  genomic data for other Foc races are currently not present in the public domain. However, since this effector gene was uniquely present in the Foc race 1 genome, and all the Foc races belong to the same VCG, we anticipate that it may detect other Foc races. Furthermore, in silico analysis has shown the absence of this effector gene in the genomes of non-pathogenic Fo (Foxy_EtdFoc: 22, 36, 191 and 136) isolated from chickpea, suggesting that this marker will differentiate pathogenic and non-pathogenic Fo isolated from chickpea plants. We have developed a novel LAMP and PCR assay which is specific and sensitive to Indian Foc race 1 and can be field deployable.

## Supplementary Information

Supplementary Information 1.

Supplementary Information 2.

## Data Availability

Genomes of the endemic Australian isolates that were sequenced for this project are available on the NCBI GenBank with Accession numbers JAMSCL000000000-JAMWEW000000000 under bioproject PRJNA846078. GenBank accession numbers are given of other genomes retrieved from the NCBI GenBank.
