# Supplementary material for: Diagnosis of Fusarium oxysporum f. sp. ciceris causing Fusarium wilt of chickpea using loop-mediated isothermal amplification (LAMP) and conventional end-point PCR
Source: Sci Rep. 2023 Feb 14;13:2640. doi: 10.1038/s41598-023-29730-6 (PMC9929042; doi:10.1038/s41598-023-29730-6)
Supplement: Supplementary file 2 — Supplementary Information 2. [file 41598_2023_29730_MOESM2_ESM.docx]

**Supplementary Figures**

MSPNKGELILPSEVLEPYVSEWSANYPTLDPSEAQLFKDWFDGLSDSSLKDYCKEPEEVSEADSCLLLLTACRLLANNQPPPSSLARRVKAWFLFTPQGDALYKILQSKDGFAVDAVIEALDRIQ

**Supplementary figure 1**. Amino acid sequence of the Foc specific molecular marker selected for the Foc diagnostic assays


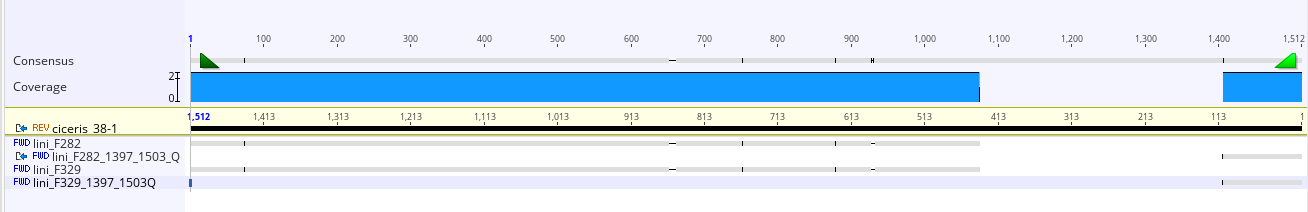


**Supplementary figure 2**. Alignment of the forward (Foc0-12f) and reverse primers (Foc0-12r) of *Fusarium* *oxysporum* f. sp. *ciceris* PCR diagnostic assay developed by Jiménez-Gasco et al. based on the impala transposon sequence with the impala transposon sequences of *Fusarium oxysporum* f. sp. *ciceris* strain 38-1 and *Fusarium oxysporum* f. sp. *lini* strains F282 and F329. As shown in the above image, the primer pair binds well to the transposon sequences in both *Fusarium* *oxysporum* f. sp. *ciceris* and *Fusarium oxysporum* f. sp. *lini*.

Ladder

negative control

Fo from tomato

Fo f. sp. l*ycopersici*

Fo f. sp. *tulipae*

Fo from natural ecosystems

Fo f. sp. *zingiberi*

Fo f. sp. *niveum*

Fo f. sp. *melonis*

Fo f. sp*. canariensis*

Fo from chickpea

Fo f. sp. *medicaginis*

Fo f. sp. *cubense*

Fo f. sp. *passiflorae*

Fo f. sp*. tracheiphilum*

Fo f. sp. *pisi* race 5

Fo f. sp. *dianthi*

Fo f. sp. *vasinfectum* (endemic)

Fo f. sp. *ciceris* (positive control)


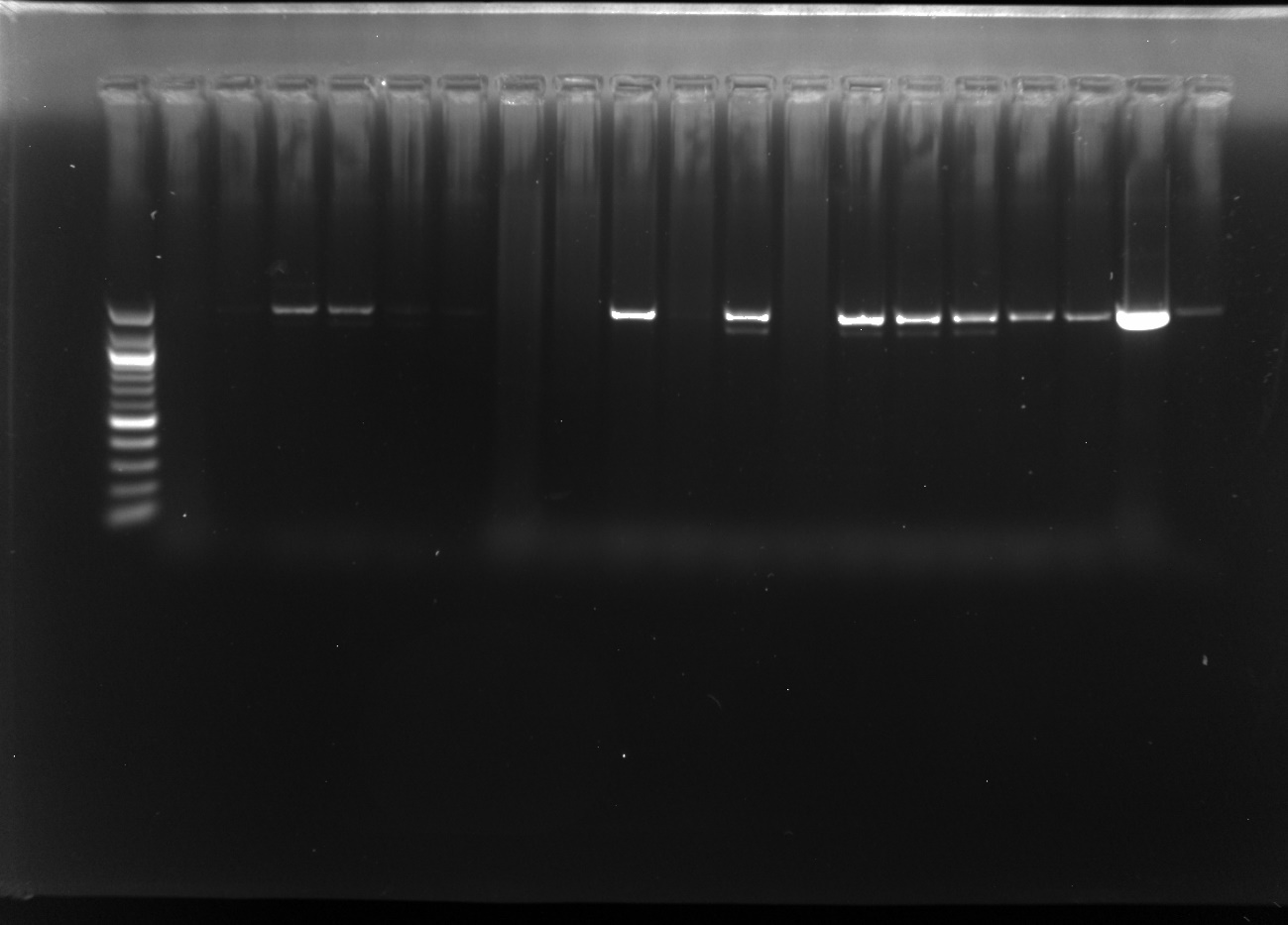


**Supplementary figure 3**. Agarose gel showing amplification products from PCR diagnostic assay developed by Jiménez-Gasco et al. based on the impala transposon sequence. There are amplifications from Foc (positive control) (lane 19) as well as from some other ff. spp. (lanes 4, 5, 10, 12, 14-18 showing a product size of 1500bp.
